# Liposomal Methylglyoxal Targets Virulence and Intracellular Persistence to Overcome Amphotericin B Resistance in *Cryptococcus neoformans*

**DOI:** 10.3390/ijms27114773

**Published:** 2026-05-26

**Authors:** Masood Alam Khan, Arif Khan, Mohd Azam, Md Zafar Iqubal, Hina Younus

**Affiliations:** 1 Department of Basic Health Sciences, College of Applied Medical Sciences, Qassim University, Buraydah 51452, Saudi Arabia; 4140@qu.edu.sa; 2 Department of Medical Laboratories, College of Applied Medical Sciences, Qassim University, Buraydah 51452, Saudi Arabia; m.aftab@qu.edu.sa; 3 Interdisciplinary Biotechnology Unit, Faculty of Life Sciences, Aligarh Muslim University, Aligarh 202002, India; mdzafariqubal789@gmail.com (M.Z.I.); hinayounus@rediffmail.com (H.Y.)

**Keywords:** antifungal therapy, cryptococcus, nanoparticle, biofilms, laccase, drug-resistant

## Abstract

Cryptococcosis, caused by *Cryptococcus neoformans,* remains a major cause of mortality in immunocompromised patients, with treatment increasingly limited by resistance and toxicity associated with Amphotericin B (Amp B). In this study, methylglyoxal (MG) was evaluated as a virulence-targeted antifungal strategy, with emphasis on its liposomal delivery. MG exhibited superior antifungal activity as compared to Amp B, demonstrating lower MIC values and significantly enhanced inhibition of biofilm formation and laccase activity, key determinants of cryptococcal pathogenicity. Notably, MG showed potent intracellular antifungal activity within macrophages, where *C. neoformans* persists. Liposomal MG (Lip-MG) markedly reduced macrophage-associated fungal burden under the tested conditions, markedly outperforming both free MG and Amp B formulations. In a leukopenic mouse model of systemic infection, liposomal MG significantly improved survival (up to ~70%) and reduced pulmonary fungal burden as compared to Amp B, which showed limited therapeutic benefit. Importantly, MG-based formulations exhibited a favorable safety profile, with significantly lower nephrotoxicity than Amp B. Collectively, these findings demonstrate that Lip-MG acts through a dual virulence-targeted activity mechanism while effectively eliminating intracellular infection. This dual action, combined with improved safety, highlights MG-based nanotherapy as a promising and translational alternative for the treatment of drug-resistant cryptococcosis.

## 1. Introduction

*Cryptococcus neoformans* remains a leading cause of morbidity and mortality worldwide, particularly among individuals with impaired cell-mediated immunity, such as patients with HIV/AIDS, hematological malignancies, or organ transplant recipients [1,2]. Unlike many opportunistic fungal pathogens, including *Candida albicans* and *Aspergillus fumigatus*, which can also cause localized infections and, in rare cases, invasive disease in immunocompetent individuals, *C. neoformans* predominantly causes disease in immunocompromised hosts with impaired cell-mediated immunity. Infections in immunocompetent individuals are comparatively uncommon and are more classically associated with *Cryptococcus gattii*, although occasional cases of *C. neoformans* infection in apparently immunocompetent individuals have been reported [3,4,5]. Importantly, *C. neoformans* can survive and replicate both intracellularly within macrophages and extracellularly in host tissues, reflecting its dual pathogenic lifestyle [6]. Following inhalation, the pathogen survives and replicates within host macrophages, which function as “Trojan horses” that facilitate dissemination, immune evasion, and long-term latency until host defenses decline [7]. Clinical manifestations range from asymptomatic or mild pulmonary infection to severe disseminated disease and fatal meningoencephalitis, depending on host immune status and fungal virulence [8,9].

Amphotericin B (Amp B) has long been regarded as the cornerstone of induction therapy for cryptococcal meningitis because of its rapid and broad-spectrum fungicidal activity. However, its clinical utility is increasingly compromised by severe nephrotoxicity, infusion-related adverse effects, and the emergence of resistant or tolerant *C. neoformans* isolates [10,11]. Recent clinical reports describing treatment failure with conventional Amp B-based regimens, even in immunocompetent patients, underscore a growing therapeutic gap [12]. Studies indicate that resistance and phenotypic tolerance can arise from alterations in ergosterol biosynthesis, including PKH3-mediated changes in membrane sterol composition, as well as enhanced biofilm formation, increased capsule thickness, and modulation of virulence-associated pathways, all of which reduce drug penetration and fungicidal efficacy [13]. Although. Amp B resistance remains relatively uncommon; its clinical consequences are profound, often leading to treatment failure and poor patient outcomes.

Given these limitations, there is growing interest in antifungal delivery systems that can enhance therapeutic efficacy while minimizing host toxicity. Liposomal formulations have emerged as one of the most successful and clinically validated nanocarrier platforms in antifungal therapy [14,15]. Liposomal Amp B represents a landmark advance, significantly reducing nephrotoxicity while maintaining antifungal activity as compared to conventional formulations [16]. Beyond toxicity reduction, liposomal antifungal drugs exhibit improved pharmacokinetics, enhanced penetration into infected tissues, and preferential uptake by phagocytic cells, such as macrophages, which play a central role in the pathogenesis and dissemination of *C. neoformans* [17]. Liposomal systems also enable controlled drug release and sustained antifungal exposure, properties that are particularly advantageous for treating deep-seated and disseminated fungal infections. Importantly, liposomes provide a versatile platform for delivering not only conventional antifungals but also nontraditional agents, including antivirulence compounds, thereby expanding therapeutic options for drug-resistant cryptococcosis [18]. In parallel, genome plasticity has emerged as an additional driver of antifungal resistance. Stress-induced aneuploidy in *C. neoformans* has been shown to confer resistance to fluconazole and 5-flucytosine through coordinated upregulation of resistance-associated genes, raising concerns that similar adaptive mechanisms could further undermine the long-term efficacy of existing antifungal agents [19]. Together, Amp B toxicity, emerging resistance, and fungal genomic adaptability highlight the urgent need for safer, more effective, and mechanistically distinct antifungal strategies.

Methylglyoxal (MG), a reactive by-product of glycolysis, has recently gained attention for its antimicrobial and anticancer properties. MG disrupts glycolytic enzymes and mitochondrial function, promoting apoptosis in cancer cells [20], and exhibits antimicrobial activity by suppressing biofilm formation and impairing stress-response systems, as demonstrated in bacterial pathogens, including *Pseudomonas aeruginosa* and *Staphylococcus aureus* [21]. Notably, nanotechnology-based delivery of MG has significantly enhanced its antifungal potential; MG-conjugated chitosan nanoparticles were previously shown to effectively treat fluconazole-resistant *C. albicans* infection [22]. However, the antifungal efficacy of MG against Amp B-resistant *C. neoformans* and its potential to target cryptococcal virulence determinants remain largely unexplored.

In the present study, we introduce a virulence-targeted, nanotechnology-enabled antifungal strategy based on MG-loaded liposomes. By combining in vitro antifungal and antivirulence assays with in vivo validation in immunocompromised mice, we demonstrate that MG-based liposomal therapy effectively inhibits fungal growth, suppresses virulence-associated factors, and protects against Amphotericin B-resistant cryptococcal infection while minimizing host toxicity. These findings position MG-loaded liposomes as a promising next-generation therapeutic platform for the treatment of drug-resistant cryptococcosis.

## 2. Results

### 2.1. Early In Vitro Screening Identifies MG as a Potent Antifungal Against C. neoformans

**Figure 1:**
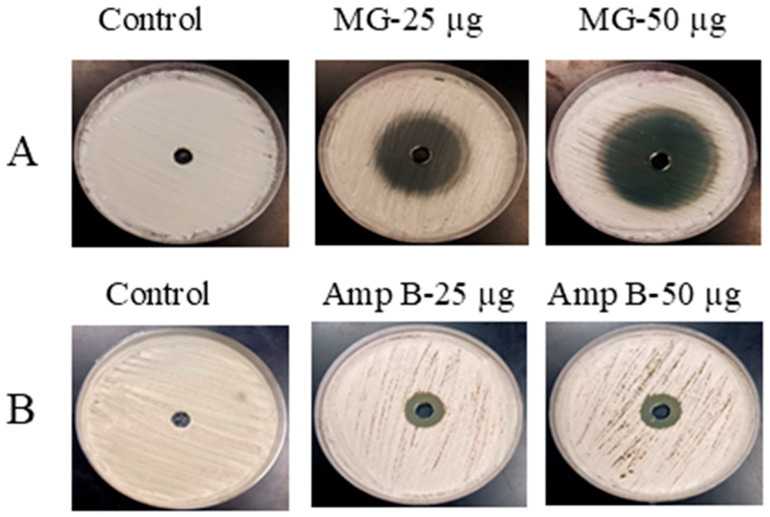
Comparative antifungal activity of MG and Amp B against *C. neoformans* assessed by agar well diffusion assay. Representative SDA plates showing zones of growth inhibition produced by (**A**) MG and (**B**) Amp B against *C. neoformans*. Wells were loaded with MG or Amp B at doses of 25 µg and 50 µg. MG produced markedly larger inhibition zones than Amp B, indicating stronger antifungal activity against the tested isolate.

The initial antifungal potential of Amp B and MG against *C. neoformans* was evaluated using the agar well diffusion assay. This screening revealed a clear difference in antifungal efficacy between Amp B and MG. MG produced pronounced and dose-dependent inhibition, as evidenced by substantially larger zones of growth suppression on SDA plates. At concentrations of 25 and 50 µg per well, MG generated inhibition zones measuring approximately 22 mm and 33 mm, respectively, demonstrating strong antifungal activity (Figure 1A). Conversely, Amp B produced much smaller zones of inhibition, measuring only 10 mm and 11 mm at the corresponding doses (Figure 1B). These results clearly indicate that MG possesses superior in vitro antifungal activity against *C. neoformans* as compared to Amp B under the tested conditions.

The Amphotericin B MIC for the present *C. neoformans* isolate was >32 µg/mL, indicating markedly reduced susceptibility (non-wild-type susceptibility) relative to published wild-type epidemiological cutoff values (ECVs), which are generally ~1 µg/mL for the *C. neoformans*/*C. gattii* species complex [23]. Because formal CLSI clinical breakpoints for *Cryptococcus* spp. are not established, this high MIC was interpreted in the context of ECV-based susceptibility distributions rather than categorical clinical resistance.

### 2.2. MG Exhibits Superior Efficacy Against Biofilm Formation in C. neoformans

**Figure 2:**
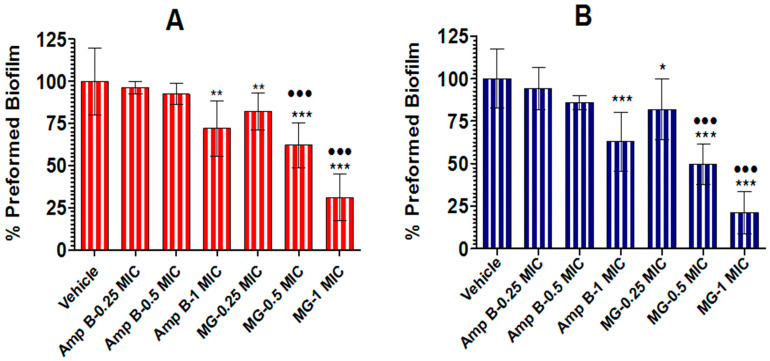
Effects of MG and Amp B on disruption of *C. neoformans* biofilms as determined by crystal violet (CV) biomass assay and XTT metabolic activity assay. Mature *C. neoformans* biofilms were established in 96-well plates and subsequently treated with Amp B or MG at 0.25×, 0.5×, and 1× MIC for 24 h. (**A**) Residual biofilm biomass was quantified by CV staining and expressed as a percentage relative to vehicle-treated controls (set as 100%). (**B**) Residual metabolic activity of treated biofilms was assessed by XTT reduction assay and similarly expressed as a percentage of untreated controls. Data are presented as mean ± SD from three independent experiments. * *p* < 0.05, ** *p* < 0.01, *** *p* < 0.001 versus vehicle control; ●●● *p* < 0.001 versus corresponding Amp B-treated group at the same concentration.

The antibiofilm activities of MG and Amp B against *C. neoformans* were quantitatively assessed using crystal violet (CV) biomass staining and XTT reduction assays (Figure 2A,B). In the CV assay (Figure 2A), Amp B exhibited only limited activity at sub-MICs, producing minimal reductions in mature biofilm biomass of approximately 5% and 8% at 0.25× and 0.5× MIC, respectively, while a moderate disruption of pre-formed biofilms (~28%) was observed at 1× MIC. In contrast, MG demonstrated significantly greater and concentration-dependent antibiofilm efficacy. MG treatment reduced established biofilm biomass by 18% at 0.25× MIC, 38% at 0.5× MIC, and 69% at 1× MIC, indicating substantially stronger disruption of mature biofilm structure than Amp B.

A similar pattern was observed in the XTT reduction assay (Figure 2B), which measures the metabolic activity of biofilm-associated cells. Amp B again showed weak effects at lower concentrations, causing only insignificant reductions of approximately 6% and 14% at 0.25× and 0.5× MIC, respectively, whereas treatment at 1× MIC produced a moderate reduction of ~37% in biofilm metabolic viability. By comparison, MG displayed markedly superior activity across all tested concentrations, reducing biofilm metabolic activity by 18% at 0.25× MIC, 50% at 0.5× MIC, and 79% at 1× MIC. Collectively, these findings clearly demonstrate that MG possesses substantially greater antibiofilm efficacy than Amp B, both in suppressing biofilm-associated metabolic activity and in effectively disrupting pre-existing *C. neoformans* biofilms.

### 2.3. MG Induces Greater Biofilm Cell Death in C. neoformans than Amp B, as Revealed by Propidium Iodide-Based Confocal Microscopy

**Figure 3:**
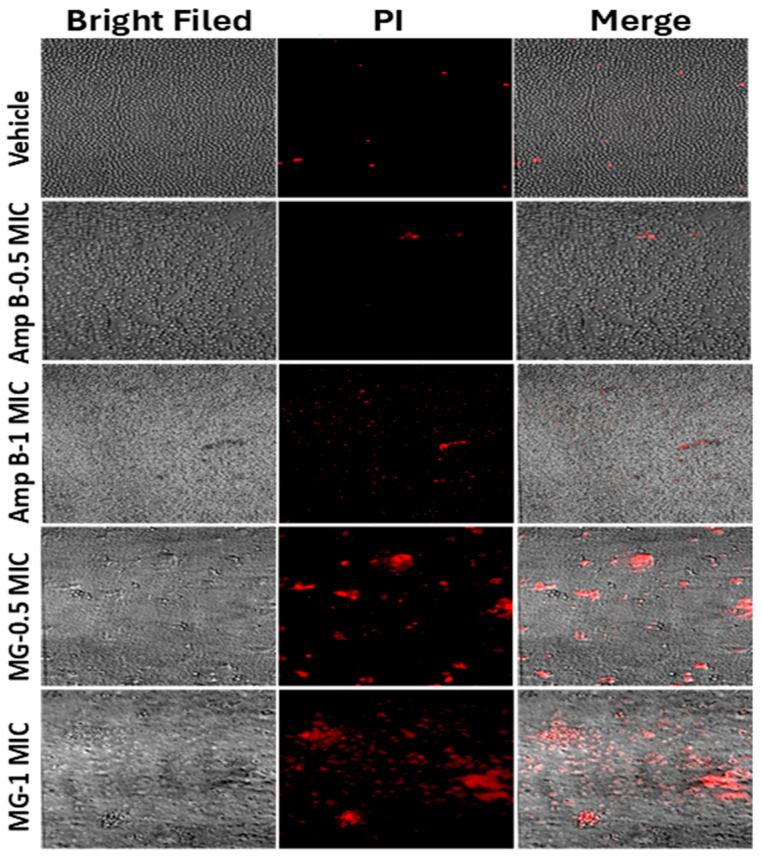
Confocal microscopic visualization of the effects of MG and Amp B on *C. neoformans* biofilm viability by using PI staining. Mature *C. neoformans* biofilms were treated with Amp B or MG at 0.5× and 1× MIC, followed by PI staining to assess fungal cell death. Columns represent bright-field images (**left**), PI fluorescence indicating membrane-compromised/dead cells (**middle**), and merged images (**right**).

The antifungal effects of MG and Amp B against *C. neoformans* biofilms were further visualized by using propidium iodide (PI)-stained confocal microscopy (Figure 3). Vehicle-treated biofilms exhibited dense and structurally intact structures in bright-field images, with minimal PI-positive fluorescence, indicating predominantly viable biofilm-associated cells. Treatment with Amp B at 0.5× MIC produced only limited antifungal activity, as evidenced by sparse PI-positive cells and relatively preserved biofilm architecture. Although Amp B at 1× MIC increased fungal cell death, as reflected by a moderate rise in red fluorescence, substantial portions of the biofilm remained structurally intact (Figure 3). In contrast, MG demonstrated markedly greater antibiofilm activity at comparable MIC levels. MG treatment at 0.5× MIC resulted in pronounced disruption of biofilm structure, accompanied by a substantial increase in PI-positive cells, indicating enhanced membrane damage and fungal death. This antifungal effect became even more pronounced at 1× MIC, where MG produced extensive red fluorescence throughout the biofilm with widespread structural collapse, suggesting extensive killing of biofilm-embedded *C. neoformans* cells. The merged images clearly confirmed that MG induced significantly greater fungal death and biofilm disruption than Amp B at equivalent concentrations.

### 2.4. Inhibition of Laccase Activity by MG and Amp B

**Figure 4:**
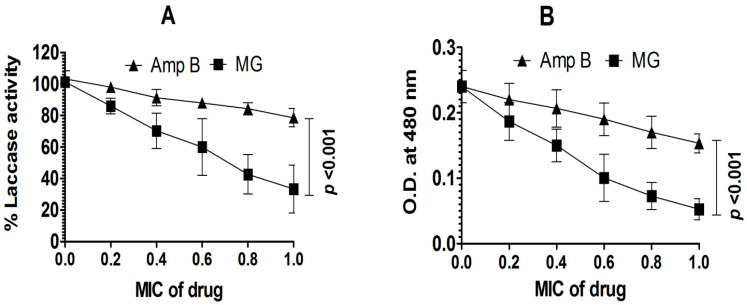
MG exhibits superior inhibition of laccase activity in *C. neoformans* as compared to Amp B. Laccase activity was assessed following exposure of *C. neoformans* cells to increasing concentrations (0–1× MIC) of MG or Amp B. (**A**) Percentage laccase activity relative to untreated control. (**B**) Corresponding oxidation of L-epinephrine was measured as optical density at 480 nm. Both drugs demonstrated dose-dependent inhibition of laccase; however, MG produced markedly greater suppression across all concentrations. Data represent mean ± SD of three independent experiments performed in triplicate. Statistical significance was determined by one-way ANOVA with Tukey’s post hoc test (*p* < 0.001, MG vs. Amp B at MIC = 1).

MG and Amp B were evaluated for their ability to inhibit laccase activity in *C. neoformans*. As shown in Figure 4, both agents caused a dose-dependent reduction in laccase function. However, MG demonstrated a substantially greater inhibitory effect on laccase activity (Figure 4A). At 1 × MIC, MG induced ~67% inhibition in laccase activity as compared to control values, whereas Amp B caused 21% decrease (*p* < 0.001). Correspondingly, L-epinephrine oxidation (measured as optical density at 480 nm) showed a parallel decline, with MG decreasing absorbance from ~0.23 to ~0.05 across the MIC range, compared to a modest reduction from ~0.23 to ~0.14 observed with Amp B (Figure 4B). Statistical analysis confirmed that MG inhibited laccase significantly more than Amp B at 1 × MIC (*p* < 0.001). These data indicate that MG exerts a pronounced suppressive effect on laccase activity, a key virulence factor in *C. neoformans*.

### 2.5. CYP-Mediated Leukocyte Depletion Confirms Immunosuppressed Model

Administration of CYP resulted in a pronounced depletion of circulating leukocytes in comparison to untreated control mice. Healthy animals displayed a mean leukocyte count of approximately 7500 ± 800 cells/mm^3^, whereas CYP-treated mice exhibited a substantial reduction in leukocyte levels, declining to 2050 ± 400 cells/mm^3^ on day 4 and 2340 ± 440 cells/mm^3^ on day 7 following treatment. This corresponds to an overall decrease of nearly 70% in total leukocyte counts. The reduction was highly significant when compared with the control group (*p* < 0.001), confirming effective induction of leukopenia. These findings validate the establishment of an immunosuppressed mouse model suitable for evaluating fungal infection and therapeutic interventions.

### 2.6. Physicochemical Characterization of Lip-Amp B and Lip-MG Formulations

Both Amp B- and MG-loaded liposomes were successfully prepared and systematically characterized to assess their physicochemical properties. Dynamic light scattering (DLS) analysis revealed mean particle diameters of 120 ± 15 nm for Lip-Amp B and 145 ± 20 nm for Lip-MG, indicating that both formulations fall within the optimal nanoscale range for systemic drug delivery (Table 1). The hydrophobic nature of Amp B facilitated strong integration into the lipid bilayer, resulting in a relatively homogeneous vesicle population with a lower polydispersity index (PDI) of 0.22 ± 0.03. In contrast, the hydrophilic MG was predominantly encapsulated within the aqueous core of the liposomes, leading to a broader size distribution and a higher PDI of 0.38 ± 0.05. Zeta potential measurements demonstrated moderately negative surface charges ranging from −18.5 to −22.3 mV, suggesting good colloidal stability of both formulations. Encapsulation efficiency varied substantially between the two drugs, with Lip-Amp B exhibiting high drug loading (~92%), whereas Lip-MG showed lower encapsulation efficiency (~38%), reflecting the inherent challenges of passively entrapping hydrophilic molecules. Overall, these findings confirm the successful formulation of stable liposomal systems and underscore the critical influence of drug physicochemical properties on liposome size uniformity and loading efficiency.

### 2.7. Marked Reduction in Macrophage-Associated Fungal Burden by Lip-MG Compared with Amp B

**Figure 5:**
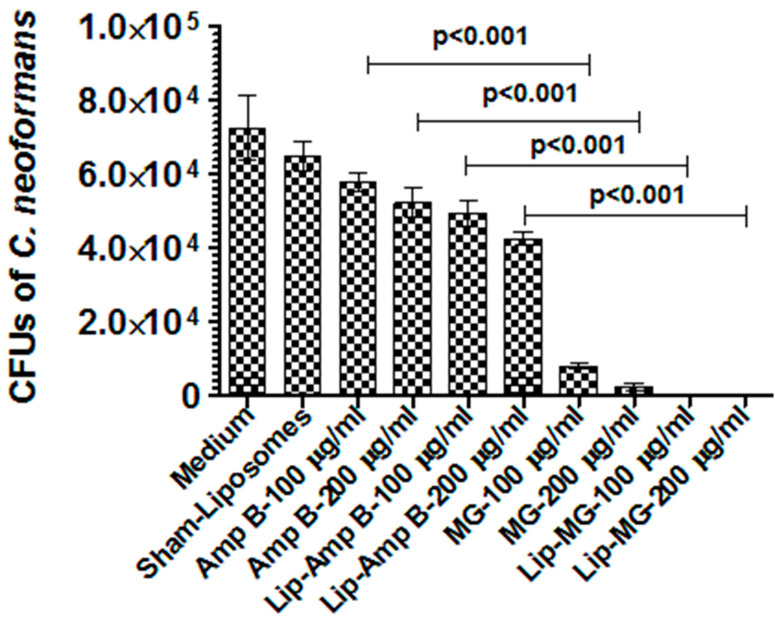
Macrophage-associated antifungal efficacy of Amp B and MG formulations against *C. neoformans*. Macrophages infected with *C. neoformans* (MOI = 2) were treated with free or Lip-Amp and MG at 100 and 200 µg/mL. After 48 h, macrophage-associated fungal burden was quantified by CFU assay. Lip-MG showed the highest efficacy with near-complete fungal clearance, followed by free MG and Lip-Amp B. Data are presented as mean ± SD (*n* = 3; *p* < 0.001).

The macrophage-associated antifungal activity of Amp B and MG, in both free and liposomal formulations, was assessed against *C. neoformans* within macrophages by quantifying CFUs. The untreated control (medium) and sham-liposome groups exhibited the highest macrophage-associated fungal burden (7.2 × 10^4^ CFUs and 6.5× 10^4^, respectively), confirming successful fungal survival within the macrophage-associated environment of the pathogen. Treatment with free Amp B produced a moderate, dose-dependent reduction in CFUs, decreasing fungal load to ~5.8 × 10^4^ and ~5.2 × 10^4^ at 100 and 200 µg/mL, respectively. Liposomal Amp B (Lip-Amp B) demonstrated improved efficacy over the free drug, further reducing CFUs to ~4.9 × 10^4^ and ~4.2 × 10^4^ at corresponding doses, indicating enhanced intracellular delivery (Figure 5). In contrast, MG showed substantially greater antifungal activity. Free MG markedly reduced macrophage-associated CFUs to ~8.0 × 10^3^ (100 µg/mL) and ~2.5 × 10^3^ (200 µg/mL). Notably, Lip-MG exhibited the most potent effect, nearly eliminating macrophage-associated fungi, with CFUs reduced to ~63 at 100 µg/mL and approaching complete clearance (~4 CFUs) at 200 µg/mL (Figure 5). All groups treated with MG formulations showed statistically significant reductions as compared to those treated with Amp B at comparable doses (*p* < 0.001), with Lip-MG demonstrating superior efficacy over all other formulations.

### 2.8. Liposomal-MG Outperforms Amp B in Protecting Leukopenic Mice from C. neoformans Infection

**Figure 6:**
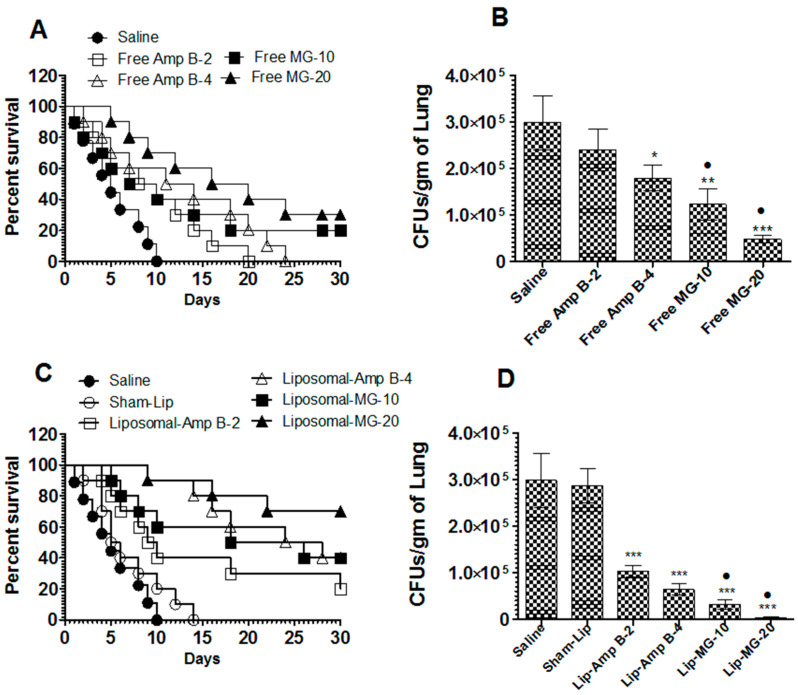
Liposomal-MG markedly improves survival and reduces fungal burden in leukopenic mice infected with *C. neoformans*, compared with free MG and Amp B formulations. (**A**) Kaplan–Meier survival curves of *C. neoformans*-infected leukopenic mice treated with free Amp B (2 and 4 mg/kg) or MG (10 and 20 mg/kg). (**B**) Lung fungal burden (CFU/g tissue) in the corresponding MG-treated groups on day 7 post-infection. Statistical significance was determined relative to saline (* *p* < 0.05, ** *p* < 0.01, *** *p* < 0.001), Free Amp B-2 vs. Free MG-10 or Free Amp B-4 vs. Free MG-20 (● *p*< 0.05) (**C**) Survival analysis of infected mice treated with Lip-Amp B (2 and 4 mg/kg) or Lip-MG (10 and 20 mg/kg). (**D**) Lung CFU counts in Lip-Amp B (2 and 4 mg/kg) or Lip-MG (10 and 20 mg/kg) treated groups. Each group contained ten mice, and the data are expressed as mean ± SD. Statistical significance was determined relative to sham-liposome controls (*** *p* < 0.001), Lip-Amp B-2 vs. Lip-MG-10 or Lip-Amp B-4 vs. Lip-MG-20 (● *p* < 0.05).

Untreated mice showed rapid disease progression, with complete mortality by ~10 days. Free Amp B produced only a modest delay in mortality, with all animals eventually succumbing by day 30, even at the higher dose (Figure 6A). Although free Amp B at 4 mg/kg modestly prolonged median survival time (MST) from 5 days in saline controls to 14.5 days, this improvement was insufficient to confer long-term protection. In comparison, free MG demonstrated a clearer dose-dependent survival benefit, with approximately ~20% survival at 10 mg/kg and ~30% survival at 20 mg/kg (*p* = 0.0714 and *p* = 0.0006; Figure 6A). These findings indicate that free MG provides better protection than free Amp B under the same experimental conditions. Consistent with survival data, saline-treated mice exhibited high pulmonary fungal loads (Figure 6B) (~3.0 × 10^5^ CFU/g). Free Amp B resulted in only a moderate reduction (~2.4 × 10^5^ and ~1.8 × 10^5^ CFU/g at 2 and 4 mg/kg, respectively). In contrast, free MG produced a significantly greater and dose-dependent reduction in fungal burden (~1.2 × 10^5^ CFU/g at 10 mg/kg and ~5.0 × 10^4^ CFU/g at 20 mg/kg), demonstrating superior antifungal efficacy compared to free Amp B (*p* < 0.05).

Liposomal delivery improved therapeutic outcomes for both agents; however, the magnitude of improvement differed substantially. Lip-Amp B achieved partial protection, with ~20% survival at 2 mg/kg and ~40% at 4 mg/kg by day 30 (Figure 6C). In contrast, Lip-MG showed markedly enhanced efficacy, with ~40% survival at 10 mg/kg and up to ~70% survival at 20 mg/kg (*p* = 0.0021 and *p* < 0.0001). Fungal burden analysis further supported these observations. While Lip-Amp B reduced lung CFUs (~1.0 × 10^5^ and ~6.4 × 10^4^ CFU/g at 2 and 4 mg/kg, respectively), Lip-MG achieved a substantially greater reduction (~3.2 × 10^4^ CFU/g at 10 mg/kg and ~4.3 × 10^3^ CFU/g at 20 mg/kg) (Figure 6D). These reductions were significantly lower than both saline and sham-liposome controls and clearly superior to Lip-Amp B.

### 2.9. MG-Based Formulations Exhibit Minimal Renal Toxicity, Whereas Amp B Exacerbates Kidney Injury

**Figure 7:**
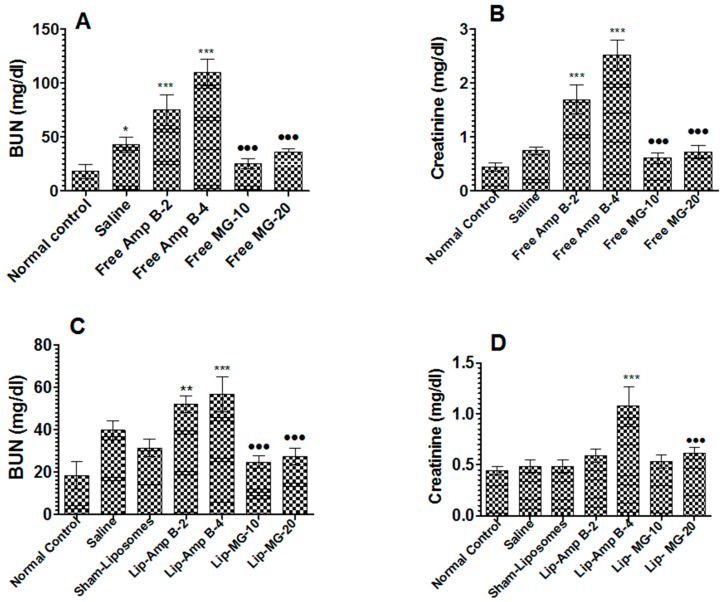
Evaluation of renal toxicity following antifungal treatments in *C. neoformans*-infected leukopenic mice. (**A**) Serum BUN levels in mice treated with free Amp B or free MG at the indicated doses. (**B**) Corresponding serum creatinine levels showing marked elevation in free Amp B-treated groups, whereas free MG-treated mice demonstrated significantly lower creatinine levels. (**C**) BUN levels in mice treated with Lip-Amp B or Lip-MG formulations. (**D**) Serum creatinine levels following Lip-Amp B or Lip-MG therapy. Each group contained ten mice, and the data are expressed as mean ± SD; statistical significance is indicated as * *p* < 0.05, ** *p* < 0.01, *** *p* < 0.001 compared with saline or sham-liposomes. ●●● *p* < 0.001, Free MG vs. Free Amp B or Lip-MG vs. Lip-Amp B at corresponding doses.

Renal toxicity was evaluated by measuring serum BUN and creatinine levels (Figure 7A–D). Infection with *C. neoformans* alone caused a moderate but significant increase in BUN, rising from ~18 mg/dL in normal controls to ~43 mg/dL in saline-treated infected mice (Figure 7A; *p* < 0.05). Treatment with free Amp B markedly exacerbated renal dysfunction in a dose-dependent manner, with BUN levels increasing to ~75 mg/dL and ~110 mg/dL at 2 and 4 mg/kg, respectively (*p* < 0.001 vs. control). In contrast, free MG exhibited a substantially improved safety profile, with significantly lower BUN levels (~25 mg/dL and ~36 mg/dL at 10 and 20 mg/kg, respectively; *p* < 0.001 vs. free Amp B). A similar trend was observed for serum creatinine (Figure 7B). Free Amp B induced a pronounced elevation in creatinine (~1.7 and ~2.5 mg/dL at 2 and 4 mg/kg, respectively), compared to ~0.7 mg/dL in infected saline controls (*p* < 0.001). In comparison, MG-treated groups maintained near-normal creatinine levels (~0.6–0.7 mg/dL), indicating minimal nephrotoxicity.

Liposomal formulations partially mitigated Amp B-induced renal toxicity (Figure 7C,D). Lip-Amp B reduced BUN levels relative to free Amp B (~52 and ~57 mg/dL at 2 and 4 mg/kg, respectively), yet values remained significantly elevated as compared to controls. In contrast, Lip-MG maintained low BUN levels (~24–27 mg/dL), comparable to the normal physiological range (*p* < 0.001 vs. Lip-Amp B) (Figure 7C). Consistently, creatinine levels (Figure 7D) were significantly elevated in the Lip-Amp B group (~1.1 mg/dL at 4 mg/kg) as compared to sham controls (~0.48 mg/dL; *p* < 0.001). In contrast, Lip-MG treatment (20 mg/kg) maintained significantly lower creatinine levels (~0.6 mg/dL), indicating superior renal safety. Overall, while liposomal encapsulation reduced Amp B-associated nephrotoxicity, MG, particularly in its liposomal form, demonstrated a markedly safer renal profile with minimal alterations in both BUN and creatinine, highlighting its superior therapeutic index.

## 3. Discussion

The present study establishes MG, particularly in its liposomal form, as a mechanistically distinct and translationally relevant therapeutic strategy against *C. neoformans* that is refractory to Amp B. Rather than relying on conventional sterol-targeting mechanisms, MG operates through a dual virulence-targeted activity that simultaneously disrupts biofilm architecture, suppresses laccase activity, and eliminates intracellular fungal reservoirs. This integrated mode of action directly addresses the emerging clinical challenge of Amp B resistance and toxicity. Although Amp B remains the cornerstone of antifungal therapy, its efficacy is increasingly undermined by adaptive fungal responses, including altered ergosterol composition, enhanced capsule formation, and biofilm-mediated tolerance [12,13]. These adaptations not only reduce drug susceptibility but also reinforce fungal persistence within host niches. Our findings highlight that targeting virulence determinants, rather than essential growth pathways, offers a fundamentally different and potentially more durable therapeutic strategy. Laccase-driven melanization and biofilm formation are central to cryptococcal survival, immune evasion, and resistance [24,25,26,27,28]. MG-mediated suppression of these processes therefore represents a critical disruption of pathogenic fitness, consistent with prior evidence that melanization reduces susceptibility to Amp B in vivo [29].

A defining advance of this study is the marked reduction in macrophage-associated fungal burden observed with MG formulations under the tested conditions. *C. neoformans* is uniquely adapted to survive and replicate within macrophages, using these cells as vehicles for immune evasion and dissemination. This intracellular niche remains a major limitation for conventional antifungals. In this context, MG, particularly when delivered via liposomes, produced a profound reduction in macrophage-associated fungal burden, markedly outperforming Amp B. This effect likely reflects enhanced cellular uptake, sustained intracellular release, and efficient targeting of pathogen-containing compartments. It is important to note, however, that macrophage infection models using J774 cells may not exclusively represent true intracellular fungal burden, as partial macrophage lysis over time can release internalized organisms into the extracellular environment [30]. Therefore, the CFU counts in this study likely reflect a composite measure of macrophage-associated fungal survival, including intracellular persistence, released fungi, and limited extracellular replication. Nevertheless, because all treatment groups were processed under identical experimental conditions with prior removal of non-phagocytosed cells, the observed reductions in CFU with MG and liposomal MG treatment reliably indicate impaired fungal survival and/or replication within the macrophage milieu. Beyond direct antifungal activity, MG-based nanotherapy may also potentiate host defense mechanisms, as suggested by previous reports of macrophage activation and cytokine induction [31]. Thus, MG appears to function at the interface of antimicrobial and host-directed therapy.

The in vivo efficacy further underscores the translational potential of this approach. In a leukopenic mouse model, free Amp B failed to confer meaningful protection, highlighting the dual burden of resistance and systemic toxicity. Lip-Amp B provided partial benefit, consistent with improved pharmacokinetics, yet remained insufficient to achieve durable control. In contrast, Lip-MG produced a striking improvement in survival and fungal clearance, achieving near-complete eradication of infection at higher doses. These outcomes emphasize that therapeutic success in cryptococcosis requires not only fungicidal activity but also effective penetration into protected niches and sustained activity within the host microenvironment.

Equally important is the markedly improved safety profile of MG. Amp B-induced nephrotoxicity remains a major clinical barrier, as reflected by significant elevations in BUN and creatinine levels [32,33,34,35]. While liposomal formulations partially mitigate this toxicity, they do not eliminate it entirely. In contrast, MG, especially in its liposomal form, maintained potent antifungal efficacy with minimal renal toxicity, indicating a substantially improved therapeutic index. While renal function markers (BUN and creatinine) were selected as primary indicators due to the well-established nephrotoxicity associated with Amphotericin B, a more comprehensive safety assessment would benefit from additional evaluations, including histopathological analysis and multi-organ toxicity profiling. These aspects were beyond the scope of the present study and will be addressed in future investigations. This balance between efficacy and safety is particularly critical in immunocompromised patients, where treatment-related toxicity can be as limiting as the infection itself.

Collectively, these findings support a conceptual shift in antifungal therapy, from pathogen-directed killing toward virulence-targeted and host-compatible intervention. By simultaneously impairing biofilm formation, melanization, and intracellular survival, MG disrupts the core survival strategies of *C. neoformans* while avoiding the toxicity associated with conventional agents. Liposomal delivery further amplifies these advantages by enhancing bioavailability, enabling intracellular targeting, and reducing off-target effects.

Looking forward, several avenues merit investigation to advance this platform toward clinical translation. Although the intravenous infection model used in this study was intentionally selected to generate a rapid, reproducible disseminated infection in leukopenic mice for consistent evaluation of systemic antifungal efficacy, survival, and fungal burden, it does not fully replicate the natural inhalational route or early pulmonary pathogenesis of *C. neoformans*. Accordingly, future studies using intranasal or inhalational infection models will be important to strengthen physiological and translational relevance. In parallel, pharmacokinetic and long-term safety studies are essential to define optimal dosing strategies. Given the neurotropism of cryptococcal infection, evaluation in central nervous system models will also be critical to establish therapeutic relevance for cryptococcal meningitis. In addition, rational combination strategies integrating MG-liposomes with reduced-dose conventional antifungals may provide synergistic efficacy while limiting resistance and toxicity. Finally, deeper interrogation of host–pathogen interactions, including immune modulation and macrophage polarization, will further clarify the dual antifungal and immunotherapeutic potential of MG. In summary, this study suggests that Lip-MG represents a promising antifungal approach with potential advantages over existing therapies. Its ability to reduce macrophage-associated fungal burden under experimental conditions, impair key virulence mechanisms, and maintain a favorable safety profile indicates its potential for further development and evaluation as a therapeutic option for drug-resistant cryptococcosis.

## 4. Materials and Methods

### 4.1. Materials

Sabouraud dextrose agar (SDA) and Sabouraud dextrose broth (SDB) were procured from Condalab (Madrid, Spain). Commercial biochemical assay kits used for the estimation of blood urea nitrogen (BUN) and creatinine were obtained from Química Clínica Aplicada (Amposta, Tarragona, Spain).

### 4.2. C. neoformans

An isolate of *C. neoformans* was obtained from the Department of Microbiology, King Fahad Hospital, Buraydah, Saudi Arabia, and maintained on Sabouraud Dextrose Agar plates. Microscopic examination showed encapsulated budding yeast cells, and urease test positivity further confirmed the identity of *C. neoformans*. In addition to microscopic examination and a positive urease test, the isolate was further identified using the VITEK^®^ system (bioMérieux, Marcy-l’Étoile, France), which enables reliable species-level classification based on biochemical profiling. The isolate also exhibited pronounced laccase activity, a well-recognized virulence-associated trait of *C. neoformans*, providing additional functional confirmation of its identity.

### 4.3. Antifungal Activity of MG and Amp B

An agar well diffusion assay was used to assess the antifungal activity of MG and Amp B against *C. neoformans* [22]. SDA plates were evenly inoculated with the yeast suspension, and 8 mm wells were created. Each well was filled with 50 µL of MG or Amp B at concentrations of 25 and 50 µg. Following incubation at 37 °C for 48 h, inhibition zones were measured in millimeters. Wells containing sterile saline served as negative controls to ensure the specificity of the antifungal effect.

The minimum inhibitory concentration (MIC) of both amp B and MG was determined by the broth macrodilution method according to the guidelines of Clinical and Laboratory Standards Institute (CLSI), Wayne (NCLLS) [36]. The final concentrations of Amp B and MG used were in the range of 1 to 250 µg/mL. Antifungal susceptibility testing was performed in small test tubes. The inoculum of *C. neoformans* was prepared in Sabouraud dextrose broth (SDB) containing 1 × 10^5^ cells per ml. The tubes were incubated at 37 °C for 48 h. The absorbance was taken at 530 nm, and the MIC was considered as the lowest drug concentration in the test tube that showed no visible growth.

### 4.4. Assessment of Methylglyoxal and Amphotericin B on Eradication of C. neoformans Biofilms by Crystal Violet Microtiter Plate and XTT Reduction Assays

The effects of MG and Amp B on *C. neoformans* biofilm formation and disruption were evaluated by using both crystal violet (CV) microtiter plate and XTT reduction assays, as previously described with minor modifications [37,38]. For biofilm eradication analysis, standardized fungal suspensions (1 × 10^6^ CFUs) were seeded into 96-well plates and incubated at 37 °C for 48 h to allow biofilm development. Following incubation, non-adherent cells were removed by washing with phosphate-buffered saline (PBS), and the established biofilms were treated with MG or Amp B at concentrations corresponding to 0.25×, 0.5×, and 1× MIC for an additional 24 h. For CV quantification, treated wells were washed with PBS, stained with 0.1% crystal violet for 15 min, rinsed thoroughly, and the bound dye was solubilized using 30% acetic acid. Biofilm biomass was quantified by measuring absorbance at 570 nm. Biofilm eradication percentages were calculated relative to untreated biofilm controls, while vehicle controls and drug-only blanks were included for background correction.

For metabolic activity assessment, the XTT reduction assay was performed using a commercial XTT assay kit (Abcam, Cambridge, UK). In the biofilm inhibition assay, fungal suspensions (100 µL; 1 × 10^6^ CFU/mL) were cultured in 96-well plates for 48 h at 37 °C to evaluate inhibition of biofilm development. After washing to remove non-adherent cells, biofilms were further exposed to the respective treatments for 24 h. Residual metabolic activity was then quantified by XTT reduction, and absorbance was recorded using a microplate spectrophotometer at 450 nm and 660 nm. Together, these complementary assays enabled quantitative assessment of both structural biomass and metabolic viability of *C. neoformans* biofilms following treatment.

### 4.5. Analysis of the Effect of MG or Amp B Treatments on C. neoformans Biofilms by Confocal Microscopy

*C. neoformans* (1 × 10^6^ CFUs) was cultured in a 96-well plate for 48 h. After gentle washing with PBS, non-adherent cells were removed, and the biofilms were exposed to MG or Amp B (0.5× and 1× MIC) for an additional 24 h. The treated biofilms were stained with propidium iodide (PI) for 30 min. After washing, the cells were analyzed by confocal microscopy using a 20× magnification objective as described earlier [18].

### 4.6. Effect of Amp B or MG on Laccase Activity in C. neoformans

*C. neoformans* was cultured in glucose-supplemented asparagine medium and incubated at 37 °C for 48 h [39]. After incubation, the yeast cells were harvested, washed twice with PBS, and adjusted to a final density of 1 × 10^7^ cells/mL. The cell suspensions were then treated with different concentrations of Amp B or MG, corresponding to 0, 0.25 MIC, and 0.5 MIC. To determine enzyme activity, ABTS (2,2′-azino-bis (3-ethylbenzothiazoline-6-sulfonic acid) was used as the chromogenic substrate. A final concentration of 1 mM ABTS was added to the suspensions, which were incubated at 37 °C for 2 h. Following incubation, cells were separated by centrifugation, and the absorbance of the resulting supernatant was measured at 420 nm to quantify laccase-mediated ABTS oxidation. Control suspensions prepared without ABTS were included to establish baseline readings.

The effects of MG and Amp B on laccase activity in *C. neoformans* were assessed by measuring the oxidation of L-epinephrine, following a modified protocol described previously [23,40]. Briefly, *C. neoformans* was cultured overnight in Sabouraud dextrose broth (SDB) at 37 °C, harvested, and washed twice with sterile PBS. A 100 µL aliquot containing approximately 1 × 10^6^ cells was incubated with 0.1 mM L-epinephrine in the presence or absence of MG or Amp B at concentrations equivalent to 0, 0.25, 0.5, and 1× MIC. The mixtures were incubated at 37 °C for 2 h. Drug-free samples served as positive controls for laccase activity. The reaction was terminated by the addition of 1 M potassium cyanide (KCN), followed by centrifugation to remove cells. The absorbance of the supernatant was then measured at 480 nm to quantify the extent of L-epinephrine oxidation, reflecting the relative laccase activity.

### 4.7. Preparation and Characterization of Liposomal Amphotericin B (Lip-Amp B) and Liposomal Methylglyoxal (Lip-MG)

Lip-Amp B was prepared by the thin-film hydration method with slight modifications [40]. Briefly, dipalmitoylphosphatidylcholine (DPPC), cholesterol, and Amp B were dissolved in chloroform–methanol (1:1, *v*/*v*) at a lipid-to-drug ratio of 10:1 (*w*/*w*). The solvent was evaporated under reduced pressure using a rotary evaporator to form a uniform lipid film, which was hydrated with sterile normal saline and probe-sonicated to obtain homogenous liposomes. Lip-MG was prepared following a dried reconstituted vesicle (DRV) approach [24,25]. DPPC and cholesterol were dissolved in chloroform–methanol (1:1, *v*/*v*), dried under vacuum to form a thin lipid layer, hydrated with 20 mM PBS (pH 7.4), sonicated, and lyophilized to generate DRVs. These were rehydrated with 2 mg/mL MG and briefly sonicated to obtain Lip-MG; unencapsulated MG was removed by centrifugation. For encapsulation efficiency, Lip-Amp B was lysed with methanol, and absorbance was measured at 412 nm against a standard Amp B curve. Lip-MG was treated with 0.5 M perchloric acid to release MG, derivatized with 1.8 mM 1,2-diaminobenzene (DAB) to form 2-methylquinoxaline, and absorbance was recorded at 336 nm. Encapsulation efficiency was >90% for Amp B and 42% for MG. Particle size and polydispersity index (PDI) were determined by dynamic light scattering (DLS) using a Malvern Zetasizer Nano. Samples were diluted 1:10 in ultrapure water, vortexed gently, equilibrated at 25 °C for 2 min, and analyzed immediately.

### 4.8. Intracellular Antifungal Activity of Amp B and MG Formulations Against C. neoformans

Murine macrophage-like J774 macrophages were seeded in 24-well Costar plates at a density of 1 × 10^6^ cells per well, with all experimental conditions performed in triplicate. Cells were cultured in Dulbecco’s Modified Eagle Medium (DMEM) supplemented with 10% fetal bovine serum and allowed to adhere and stabilize for 24 h under standard incubation conditions. Following stabilization, macrophages were infected with *C. neoformans* at a multiplicity of infection (MOI) of 2 to facilitate controlled phagocytic uptake. After 6 h of infection, cells were gently washed with 0.1 M phosphate-buffered saline (pH 7.4) to remove extracellular, non-phagocytosed yeast cells, ensuring that only internalized organisms were retained within the macrophages. Subsequently, infected macrophages were treated with different formulations, including free Amp B, Lip-Amp B, free MG, and Lip-MG, each at concentrations of 100 and 200 µg/mL. These treatments were applied to assess their intracellular antifungal activity. Cells were then incubated for an additional 48 h to allow sufficient interaction between the antifungal agents and the internalized fungal cells. At the end of the treatment period, macrophages were lysed using 1% Triton X-100 to release phagocytosed yeast cells. The resulting lysates were serially diluted in phosphate-buffered saline, and 100 µL aliquots of each dilution were plated onto SDA plates. Following incubation, fungal burden was determined by counting colony-forming units (CFUs). Final CFU values were calculated by multiplying the number of colonies by the corresponding dilution factor, as previously described [21].

### 4.9. Experimental Animals, Ethics Approval, and Husbandry Conditions

All animal experiments were performed in strict accordance with the ARRIVE (Animal Research: Reporting of In Vivo Experiments) Guidelines and complied with institutional as well as international regulations governing the care and use of laboratory animals. Ethical clearance for the study was obtained from the Committee of Research Ethics, Deanship of Graduate Studies and Scientific Research, Qassim University, Saudi Arabia (Approval No. #24-94-03; dated 9 June 2024). Male Swiss albino mice, aged 10–12 weeks and weighing 25–30 g, were procured from the Animal House Facility of the College of Applied Medical Sciences, Qassim University. A total of 72 mice were included in the study and were acclimatized for one week prior to the initiation of experimental procedures. Animals were housed in sterile polypropylene cages under controlled environmental conditions (22 ± 2 °C temperature; 50–60% relative humidity; 12 h light/dark cycle). Standard pellet diet and filtered drinking water were provided ad libitum. Husbandry practices were maintained under specific pathogen-free (SPF) conditions with routine monitoring of animal health status. For all invasive procedures, mice were anesthetized via intraperitoneal administration of a ketamine–xylazine mixture (90 mg/kg ketamine and 10 mg/kg xylazine) to minimize pain and procedural distress. Humane endpoints were predefined in accordance with ARRIVE recommendations. Animals exhibiting severe morbidity, impaired mobility, respiratory distress, or >20% body weight loss were humanely euthanized. At designated experimental endpoints, mice were sacrificed by cervical dislocation under deep anesthesia, ensuring rapid and humane euthanasia consistent with approved ethical and ARRIVE guideline protocols.

### 4.10. Immune Suppression in Mice

Mice were injected with Cyclophosphamide (CYP) (250 mg/kg) through the intraperitoneal route as reported earlier [18]. In order to assess the systemic immunosuppression, the leukocytes were counted on days 4 and 7 post-CYP administration.

### 4.11. Mouse Model of C. noeoformans Infection

To establish systemic cryptococcal infection, mice were challenged with *C. neoformans* following immunosuppression with CYP. On day 4 after CYP administration, each mouse received an intravenous inoculum of *C. neoformans*. A fungal suspension containing 7 × 10^5^ CFUs per 200 µL was prepared in sterile saline and was delivered via the lateral tail vein to ensure uniform systemic dissemination [18].

### 4.12. Treatment of C. neoformans-Infected Mice with Free and Liposomal Formulations of Amp B or MG

Following the systemic cryptococcal infection, mice were allocated to treatment groups receiving either MG or Amp B in free or liposomal formulations. Free MG and Lip-MG were administered at 10 and 20 mg/kg, whereas free and Lip-Amp B were given at 2 and 4 mg/kg. All treatments were delivered once daily through the intraperitoneal route for five consecutive days, beginning after *C. neoformans* infection. Mice were divided into five groups and each group contained ten mice: (1) Saline, (2) Free Amp B-2 mg/kg, (3) Free Amp B-4 mg/kg (4) Free MG-10 mg/kg, and (5) Free MG-20 mg/kg, Another set of experiment also comprising six groups (ten mice in each group), including (1) Saline, (2) Sham-Lip, (3) Liposomal-Amp B-2 mg/kg, (4) Liposomal-Amp B-4 mg/kg, (5) Liposomal-MG-10 mg/kg, and (6) Liposomal-MG-20 mg/kg. Animals were monitored closely on a daily basis for survival and clinical signs of disease for a total period of 30 days post-infection. To assess the severity of infection and therapeutic efficacy at the tissue level, three mice from each treatment group were euthanized on day 7 post-infection. Lung tissues were aseptically excised, weighed, and homogenized in cold PBS. The resulting homogenates were serially diluted and cultured on SDA plates. After incubation, fungal colonies were enumerated and expressed as CFUs per gram of lung tissue, serving as a quantitative measure of fungal burden [18].

### 4.13. Assessment of Renal Toxicity in Mice Treated with Amp B and MG Formulations

The renal safety of Amp B and MG formulations was assessed by analyzing kidney function biomarkers in serum samples [18]. To this end, blood was collected from three mice per treatment group under light anesthesia through the retro-orbital puncture. The samples were allowed to clot and were subsequently centrifuged at 2000× *g* for 10 min at 4 °C to obtain clear serum. Renal function was evaluated by measuring blood urea nitrogen (BUN) and creatinine concentrations using commercially available colorimetric assay kits, following the manufacturers’ protocols.

### 4.14. Statistical Analysis

The survival data were analyzed using the Kaplan–Meier survival curve, and statistical differences between groups were assessed with the log-rank test. Data related to fungal burden and biofilm formation were evaluated using a One-way analysis of variance (ANOVA). To further identify group-wise differences, this was followed by Tukey’s post hoc test using GraphPad Prism software, version 6.0 (La Jolla, CA, USA).

## Figures and Tables

**Table 1:** Physicochemical properties of Lip-Amp B and Lip-MG.

Parameter	Lip-Amp B	Lip-MG	Interpretation
Mean Particle Size (nm)	120 ± 15	145 ± 20	Optimal nanoscale range (50–200 nm), hydrophilic MG results in slightly larger vesicles due to aqueous-core loading.
Polydispersity Index (PDI)	0.22 ± 0.03	0.38 ± 0.05	Lower PDI indicates higher uniformity.
Zeta Potential (mV)	−18.5 ± 1.2	−22.3 ± 1.6	Moderately negative charge supports colloidal stability.
Entrapment Efficiency (EE%)	92%	38%	Hydrophobic Amp B shows higher incorporation; MG exhibits lower encapsulation.

## Data Availability

The original contributions presented in this study are included in the article. Further inquiries can be directed to the corresponding author.
